# LGBTQ+ status and sex of record in Veterans with post-traumatic stress disorder: demographics, comorbidities, and outpatient encounters

**DOI:** 10.3389/fpubh.2024.1487866

**Published:** 2025-01-06

**Authors:** Terri Elizabeth Workman, Joseph L. Goulet, Cynthia A. Brandt, Melissa Skanderson, John O’Leary, Kirsha S. Gordon, Qing Treitler-Zeng

**Affiliations:** ^1^Washington DC VA Medical Center, Washington, DC, United States; ^2^Biomedical Informatics Center, The George Washington University, Washington, DC, United States; ^3^VA Connecticut Healthcare System, West Haven, CT, United States; ^4^Yale School of Medicine, Yale University, New Haven, CT, United States

**Keywords:** Veterans, sex of record, PTSD - post-traumatic stress disorder, LGBTQ+, comorbidities

## Abstract

**Objectives:**

This study aims to analyze differences between lesbian, gay, bisexual, transgender, and queer (LGBTQ+) and non-LGBTQ+ Veterans with post-traumatic stress disorder (PTSD) in terms of demographics, comorbidities, and medical care usage, including differences by sex of record, including separate analyses for transgender and non-transgender Veterans.

**Methods:**

Chi-square, *t*-test, ANOVA Welch one-way testing, and absolute standardized difference analyses were conducted on a cohort of 277,539 Veterans diagnosed with PTSD.

**Results:**

The study found significant differences, particularly concerning positive LGBTQ+ status and sex of record. There were significant differences found in age, marital status, and medical care usage, as well as pain, mental health, and substance use disorder diagnoses. Differences in having experienced military sexual trauma, crime, or maltreatment were especially significant, with increased percentages among LGBTQ+ individuals, and sex of record females. In separate analyses, there were similar differences among transgender and non-transgender Veterans, with similar increased risks for sex of record females.

**Conclusion:**

Our findings suggest an intersectionality of LGBTQ+ status and sex of record in the context of PTSD. These findings may help guide future research, policy, and interventions.

## Introduction

Post-traumatic stress disorder (PTSD) is a condition people may develop after experiencing or witnessing a life-threatening event (1). It is defined as “exposure to actual or threatened death, serious injury, or sexual violence” through direct experience, witnessing, or finding that a close associate, friend, or family member has experienced this type of event (2). It is characterized by recurrent, intrusive memories and dreams, dissociative reactions such as flashbacks, psychological distress from cues that are reminiscent of the event, and other attributes (2). PTSD can last for months or years with lasting symptoms.

Approximately 12 million people in the United States experience PTSD each year; this includes 4% of men and 8% of women (3). Individuals who have experienced a traumatic brain injury (TBI) are at greater risk, especially military personnel (4). PTSD is also associated with other mental health disorders, particularly depression, anxiety (5), and substance abuse disorders (5–7). Substance abuse co-occurs in approximately 40% of individuals with PTSD (8–10). Pain is also associated with PTSD (11–13), including in Veterans with PTSD (14). Veterans often experience a higher rate of PTSD, particularly among women, and rates vary by deployment (15, 16). Even when combat is a factor, PTSD can be a complex condition, affected by other traumatic experiences, particularly for women (17–19).

Previous research has examined PTSD with respect to sexual orientation and gender identity. Lesbian, gay, bisexual, transgender, and queer (LGBTQ+) individuals are 2.2 times more likely to experience PTSD than their heterosexual/cisgender peers (20), with rates among lesbian/bisexual/gay individuals as high as 47.6%, and rates among transgender individuals ranging from 17.8 to 42.0% (21–26). LGBTQ+ individuals have an increased prevalence of PTSD-connected events such as physical and sexual assault, and child abuse (27). Transgender individuals are 2.52 times more likely, and bisexual individuals are 2.44 times more likely, to experience PTSD than other LGBTQ+ subgroups (20).

Studies addressing PTSD in Veterans in terms of LGBTQ+ status indicate PTSD may be more prevalent among LGBTQ+ Veterans than non-LGBTQ+ Veterans (28, 29). LGBTQ+ Veterans experience several kinds of traumatic and stressful events, including assault, verbal abuse, discrimination, social isolation, shame, and microaggressions such as slights and inaccurate pronoun usage (30). PTSD is more prevalent among transgender Veterans than matched non-transgender Veterans (31, 32). Transgender Veterans are also more likely to experience PTSD as well as depression, suicidality, serious mental illness, military sexual trauma (MST), incarceration, and homelessness (32). Veterans with minoritized sexual orientation are 2.35 times more likely to have “likely PTSD” (33). PTSD, depression, anxiety, binge eating, problematic anger, multiple somatic symptoms, and insomnia are more prevalent among lesbian, gay, and bisexual Veterans and service members (34).

Less is known about the prevalence and effects of PTSD among LGBTQ+ Veterans who are women. The risk for PTSD among women LGBTQ+ Veterans may be multiplicative, meaning the combined effect of sexual orientation and gender is larger than the product of their individual effects. Gorman et al. (35) found that, while sexual minority female Veterans have comparable PTSD rates as their heterosexual peers, deployment-related stressors were less likely to be associated with PTSD. While there are some previous studies on transgender Veterans with PTSD, there are knowledge gaps regarding transgender Veterans compared to other LGBTQ+ and non-LGBTQ+ individuals with PTSD. Data that can potentially address these and other knowledge gaps can be found in the U.S. Department of Veterans Affairs (VA) electronic health records (EHRs).

In this study of Veterans with PTSD, our objectives were (1) to analyze and describe the differences between LGBTQ+ and non-LGBTQ+ Veterans in terms of demographics, comorbidities, and medical care usage; and (2) to analyze differences between male and female LGBTQ+ and non-LGBTQ+ Veterans using sex of record. Because sex of record values in VA EHR data indicate birth sex and not gender or gender identity and noting that the experience of transgender Veterans with PTSD may be different, we also conducted the same analyses for transgender Veterans in the cohort. The specific gender identities and sexual orientations of the transgender Veterans in the cohort are unknown. We included selected comorbidities in order to gain greater insight in terms of previous research. We hypothesized there would be differences between LGBTQ+ Veterans and non-LGBTQ+ Veterans, as well as between male and female sex of record, and transgender Veterans vs. non-transgender Veterans. These results could help characterize the PTSD experience for all Veterans and possibly provide insight for future interventions.

## Methods

### Data/cohort

Veterans Affairs EHR records, stored within the VA central data warehouse (CDW), served as the data source for this cross-sectional study, and we accessed these data within the secure VA Informatics and Computing Infrastructure (VINCI) research platform (36).

Using an established cohort from a previous study (37) that classified patients as LGBTQ+ or non-LGBTQ+, we identified those who had received two or more PTSD diagnoses (ICD-10 F43.1) in the 5 years between 1 January 2017 and 31 December 2021 in outpatient care encounters. We limited the PTSD diagnosis count to a maximum of one per day, to ensure individual diagnoses were at least 1 day apart. Transgender Veterans (with two or more PTSD diagnoses) were identified in an additional process, using both natural language processing (NLP) (37) and ICD codes to determine transgender status.

### Covariates

The baseline covariates were demographics (age at first diagnosis of PTSD, race, ethnicity, marital status, and sex of record) and selected comorbidities of interest based on the International Classification of Diseases (ICD) 9th and 10th revision codes, or clinical structural data element [pain diagnosis, alcohol and drug use, mental illness (e.g., bipolar disorder), traumatic brain injury (TBI), violent crime/maltreatment, and MST], and medical care usage (i.e., outpatient visits). ICD codes identifying comorbid diagnoses were previously established by the VA Musculoskeletal Diagnosis Cohort (38), Headache Centers of Excellence (39), the VA Northeast Program Evaluation Center, and Kaiser Permanente, with the exception of violent crime/maltreatment. In the case of violent crime/maltreatment, we used ICD-9 codes 995.54, 995.80–85, E960-E966, E968-, and E969-and ICD-10 codes X92-X99; Y00-Y04, Y08-Y09, Z04.41, Z65.4, Z91.41-, and T74-. For MST, we used EHR flag administrative data.

### Statistical analysis

For this descriptive study, chi-square testing was applied to the categorical covariates, *t*-test was applied to continuous covariates in comparing two samples, and ANOVA Welch one-way testing was applied to continuous covariates in comparing four samples to detect statistically significant differences by (1) LGBTQ+ status, (2) LGBTQ+ status and sex of record, (3) transgender status, and (4) transgender status and sex of record. As an additional significance test, we calculated absolute standardized difference (ASD). An ASD > 10% indicates an imbalance between two groups (40, 41) considered clinically meaningful. A difference with a *p*-value of <0.05 and ASD of >10 was considered significant. Statistical analyses were conducted with SAS, version 9.4., and Python (version 3.6.10) for ASD.

## Results

There were 277,539 patients with PTSD between calendar years 2017 and 2021, of whom 76,080 (27.4%) were LGBTQ+, 56,986 (20.5%) were female, 220,553 (79.5%) were male, and 17,278 (6.2%) were transgender Veterans.

### LGBTQ+ status in Veterans with PTSD

There are several significant statistical differences between LGBTQ+ Veterans compared to non-LGBTQ+ Veterans (Table 1). LGBTQ+ Veterans compared to non-LGBTQ+ Veterans were less likely to be married (39.7% vs. 49.5%) and more likely to be female (32.9% vs. 15.9%) and have a diagnosis for drug use disorder (36.7% vs. 29.8%), bipolar disorder (22.1% vs. 14.8%), general anxiety disorder (20.6% vs. 16.2%), and major depressive disorder (67.0% vs. 57.9%). LGBTQ+ Veterans were also more likely to be victims of violent crime/maltreatment (10.4% vs. 5.4%), and MST (40.4% vs. 23.2%). LGBTQ+ Veterans also had more outpatient care encounters on average.

**Table 1:** Demographics, comorbidities, and mean outpatient encounters by LGBTQ+ Veterans with PTSD vs. non-LGBTQ+ Veterans with PTSD.

	LGBTQ+ % (*n*)	Non-LGBTQ+ % (*n*)	*P*-value	ASD
	(*N* = 76,080)	(*N* = 201,459)		
Age, mean, (SD)	50.6 (15.1)	52.0 (15.5)	<0.0001	9
Sex of record				
M	67.1 (51074)	84.1 (169479)		**40**
F	32.9 (25006)	15.9 (31980)		**40**
Marital status			<0.0001	
Married	39.7 (30204)	49.5 (99779)		**20**
Never Married	25.9 (19714)	16.3 (32769)		**24**
Divorced/Other	34.4 (26162)	34.2 (68911)		0
Ethnicity			<0.0001	
Hispanic or Latino	10.5 (7972)	9.1 (18235)		5
Not Hispanic or Latino/Unknown	89.5 (68108)	90.9 (183224)		5
Race			0.2389	
Black/African American	24.5 (18650)	24.8 (49918)		1
White	64.6 (49115)	64.2 (129368)		1
Other	10.9 (8315)	11.0 (22173)		0
Pain diagnosis				
Abdominal and bowel pain	45.5 (34606)	40.7 (82059)	<0.0001	10
Back pain	69.1 (52549)	68.2 (137467)	<0.0001	2
Headache	35.8 (27259)	32 (64527)	<0.0001	8
Musculoskeletal chest pain	39.9 (30390)	37.5 (75618)	<0.0001	5
Neck pain	37.6 (28621)	35.2 (70940)	<0.0001	5
Chronic migraine	2.9 (2192)	2.3 (4740)	<0.0001	3
Tension headache	4.5 (3404)	3.8 (7677)	<0.0001	3
Orofacial ear and temporomandibular pain	11.0 (8388)	9.1 (18296)	<0.0001	6
Alcohol use disorder	41.9 (31893)	37.9 (76296)	<0.0001	8
Drug use disorder	36.7 (27892)	29.8 (60034)	<0.0001	**15**
Bipolar disorder	22.1 (16785)	14.8 (29858)	<0.0001	**19**
General anxiety disorder	20.6 (15678)	16.2 (32717)	<0.0001	**11**
Major depressive disorder	67 (50973)	57.9 (116611)	<0.0001	**19**
Traumatic brain injury	10.6 (8050)	10.6 (21371)	0.8358	0
Violent crime/maltreatment	10.4 (7933)	05.4 (10950)	<0.0001	**19**
Military sexual trauma	40.4 (30705)	23.2 (46800)	<0.0001	**37**
Outpatient visits, mean (SD)	57.6 (44.1)	47.8 (39.1)	<0.0001	**24**

Bold text indicates significant ASD values.

### LGBTQ+ status and sex of record in Veterans with PTSD

Among LGBTQ+ Veterans, females compared to males were on average significantly younger (44.9 vs. 53.4 years old), less likely to be married (29.5% vs. 44.7%), more likely to be Black/African American (28.1% vs. 22.8%), to have had a diagnosis for abdominal and bowel pain (49.4% vs. 43.6%), headache (41.8% vs. 32.9%), chronic migraine (5.0% vs. 1.8%), orofacial ear and temporomandibular pain (13.7% vs. 9.7%), and to have experienced violent crime/maltreatment (17.5% vs. 6.9%) or MST (69.1% vs. 26.3%). Male LGBTQ+ Veterans with PTSD were more likely to be white (66.9% vs. 59.8%) and have a diagnosis of alcohol (47% vs. 31.5%) or drug use disorders (40.6% vs. 28.6%) (Table 2). Similar results were observed among non-LGBTQ+ Veterans, with a few notable exceptions. Non-LGBTQ+ female Veterans were more likely to have a diagnosis of tension headache (5.9% vs. 3.4%), bipolar disorder (20.2% vs. 13.8%), general anxiety disorder (21.1% vs. 15.3%), and major depressive disorder (67.6% vs. 56.1%) than non-LGBTQ+ male Veterans. Non-LGBTQ+ male Veterans were more likely to have a diagnosis for TBI (11.4% vs. 6.6%) than non-LGBTQ+ female Veterans.

**Table 2:** Demographics, comorbidities, and mean outpatient encounters by LGBTQ+ status and sex of record of Veterans with PTSD.

	LGBTQ+			non-LGBTQ+			*p*-value*
	Female	Male	ASD	Female	Male	ASD	
% (*n*)	25,006	51,074		31,980	169,479		
Age, mean, (SD)	44.9 (12.9)	53.4 (15.3)	**60**	45.3 (12.5)	53.2 (15.7)	**56**	<0.0001
Marital status							<0.0001
Married	29.5 (7377)	44.7 (22827)	**32**	33.1 (10599)	52.6 (89180)	**40**	
Never married	33.2 (8296)	22.4 (11418)	**24**	22.7 (7249)	15.1 (25520)	**20**	
Divorced/Other	37.3 (9333)	32.9 (16829)	9	44.2 (14132)	32.3 (54779)	**25**	
Ethnicity							<0.0001
Hispanic or Latino	10.8 (2709)	10.3 (5263)	2	8.8 (2816)	9.1(15419)	1	
Not Hispanic or Latino/Unknown	89.2 (22297)	89.7 (45811)	2	91.2 (29164)	90.9 (154060)	1	
Race							<0.0001
Black/African American	28.1 (7033)	22.8 (11617)	**12**	34.5 (11046)	22.9 (38872)	**26**	
White	59.8 (14955)	66.9 (34160)	**15**	53.4 (17067)	66.3 (112301)	**27**	
Other	12.1 (3018)	10.4 (5297)	5	12.1 (3867)	10.8 (18306)	4	
Pain diagnosis							
Abdominal and bowel pain	49.4 (12341)	43.6 (22265)	**12**	47.1 (15052)	39.5 (67007)	**15**	<0.0001
Back Pain	69.0 (17244)	69.1(35305)	0	66.5 (21259)	68.6 (116208)	4	<0.0001
Headache	41.8 (10459)	32.9 (16800)	**19**	41.5 (13271)	30.2 (51256)	**24**	<0.0001
Musculoskeletal chest pain	36.6 (9147)	41.6 (21243)	10	34.0 (10877)	38.2 (64741)	9	<0.0001
Neck pain	40.4 (10092)	36.3 (18529)	8	39.3 (12565)	34.4 (58375)	10	<0.0001
Chronic migraine	5.0 (1255)	1.8 (937)	**18**	5.5 (1742)	1.8 (2998)	**20**	<0.0001
Tension headache	5.9 (1468)	3.8 (1936)	10	5.9 (1876)	3.4 (5801)	**12**	<0.0001
Orofacial ear and temporomandibular pain	13.7 (3431)	9.7 (4957)	**13**	13.1 (4193)	8.3 (14103)	**16**	<0.0001
Alcohol use disorder	31.5 (7879)	47.0 (24014)	**32**	22.5 (7195)	40.8 (69101)	**40**	<0.0001
Drug use disorder	28.6 (7152)	40.6 (20740)	**25**	20.0 (6382)	31.7 (53652)	**27**	<0.0001
Bipolar disorder	24.8 (6199)	20.7 (10586)	10	20.2 (6445)	13.8 (23413)	**17**	<0.0001
General anxiety disorder	22.4 (5612)	19.7 (10066)	7	21.1 (6736)	15.3 (25981)	**15**	<0.0001
Major depressive disorder	69.8 (17465)	65.6 (33508)	9	67.6 (21606)	56.1 (95005)	**24**	<0.0001
Traumatic brain injury	8.6 (2147)	11.6 (5903)	10	6.6 (2104)	11.4 (19267)	**17**	<0.0001
Violent crime/maltreatment	17.5 (4385)	06.9 (3548)	**33**	15.2 (4855)	03.6 (6095)	**41**	<0.0001
Military sexual trauma	69.1 (17280)	26.3 (13425)	**95**	70.6 (22582)	14.3 (24218)	**139**	<0.0001
Outpatient visits, mean (SD)	55.4 (39.3)	58.6 (46.2)	7	50.6 (35.3)	47.3 (39.7)	9	<0.0001

* *p-value across all four groups. Bold text indicates significant ASD values.*

### Transgender status in Veterans with PTSD

Transgender Veterans with PTSD compared to non-transgender Veterans with PTSD were younger (49.7 vs. 51.7 years old), less likely to be married (37.2% vs. 47.5%), more likely to have had a diagnosis of bipolar disorder (22.7% vs. 16.4%), major depressive disorder (67.2% vs. 59.9%), drug use disorder (38.2% vs. 31.3%), and to have experienced violent crime/maltreatment (10.8% vs. 6.5%), and MST (40% vs. 27.1%) (Table 3). They also had higher outpatient visits (62.5 vs. 49.7).

**Table 3:** Demographics, comorbidities, and mean outpatient encounters by transgender Veterans with PTSD vs. non-transgender Veterans with PTSD.

	Transgender % (*n*)	Non-transgender % (*n*)	*P*-value	ASD
	(*N* = 17,278)	(*N* = 260,261)		
Age, mean, (SD)	49.7(14.8)	51.7(15.4)	<0.0001	**13**
Sex of record			<0.0001	
M	68.2 (11788)	80.2 (208765)		**28**
F	31.8 (5490)	19.8 (51496)		**28**
Marital status				
Married	37.2 (6429)	47.5 (123554)		**21**
Never married	24.8 (4277)	18.5 (48206)		**15**
Divorced/Other	38.0 (6572)	34.0 (88501)		8
Ethnicity			<0.0001	
Hispanic or Latino	8.5 (1471)	9.5 (24736)		3
Not Hispanic or Latino/Unknown	91.5 (15807)	90.5 (235525)		3
Race			<0.0001	
Black/African American	20.8 (3590)	25.0 (64978)		10
White	68.2 (11784)	64.0 (166699)		9
Other	11.0 (1904)	11.0 (28584)		0
Pain diagnosis				
Abdominal and bowel pain	44.7 (7730)	41.9 (108935)	<0.0001	6
Back pain	68.6 (11847)	68.5 (178169)	0.7649	0
Headache	35.0 (6052)	32.9 (85734)	<0.0001	4
Musculoskeletal chest pain	39.0 (6733)	38.1 (99275)	0.0308	2
Neck pain	37.7 (6509)	35.8 (93052)	<0.0001	4
Chronic migraine	3.0 (522)	2.5 (6410)	<0.0001	3
Tension headache	4.4 (756)	4.0 (10325)	0.0079	2
Orofacial ear and temporomandibular pain	11.1 (1913)	9.5 (24771)	<0.0001	5
Alcohol use disorder	42.0 (7250)	38.8 (100939)	<0.0001	6
Drug use disorder	38.2 (6602)	31.3 (81324)	<0.0001	**15**
Bipolar disorder	22.7 (3919)	16.4 (42724)	<0.0001	**16**
General anxiety disorder	21.2 (3665)	17.2 (44730)	<0.0001	10
Major depressive disorder	67.2 (11615)	59.9 (155969)	<0.0001	**15**
Traumatic brain injury	11.0 (1892)	10.6 (27529)	0.1231	1
Violent crime/maltreatment	10.8 (1862)	06.5 (17021)	<0.0001	**15**
Military sexual trauma	40.0 (6913)	27.1 (70592)	<0.0001	**28**
Outpatient visits, mean (SD)	62.5(46.1)	49.7(40.2)	<0.0001	**29**

Bold text indicates significant ASD values.

### Transgender status and sex of record in Veterans with PTSD

Veterans recorded as female sex of record had significantly higher percentages of abdominal and bowel pain, headache, chronic migraine, and orofacial ear and temporomandibular pain diagnoses in both transgender and non-transgender groups (Table 4). Transgender female sex of record Veterans had a significantly higher percentage of neck pain (41.9% vs. 35.7%) than transgender male sex of record Veterans. Non-transgender female sex of record Veterans had a significantly higher percentage of tension headaches (5.9% vs. 3.5%) than non-transgender male sex of record Veterans. Female sex of record also had higher percentages of violent crime/maltreatment and MST in both transgender and non-transgender groups. For alcohol and drug use disorder, male sex of record had higher percentages in both transgender and non-transgender groups. Transgender Veterans had more outpatient visits than their non-transgender counterparts, but among transgender Veterans, there was no difference by sex of record. A higher percentage of Veterans recorded as male sex of record in both transgender and non-transgender groups were married. Non-transgender male Veterans were more likely to have a diagnosis of TBI (11.4% vs. 7.3%) than non-transgender female Veterans.

**Table 4:** Demographics, comorbidities, and mean outpatient encounters by transgender status and sex of record of Veterans with PTSD.

	Transgender			Non-transgender			*p*-value*
	Female	Male	ASD	Female	Male	ASD	
% (*n*)	5,490	11,788		51,496	208,765		
Age, mean, (SD)	45.6(13.4)	51.6(15.0)	**42**	45.1 (12.6)	53.4 (15.6)	**59**	<0.0001
Marital status							<0.0001
Married	27.6 (1515)	41.7 (4914)	**30**	32.0 (16461)	51.3 (107093)	**40**	
Never married	30.8 (1693)	21.9 (2584)	**20**	26.9 (13852)	16.5 (34354)	**26**	
Divorced/other	41.6 (2282)	36.4 (4290)	**11**	41.1 (21183)	32.3 (67318)	**19**	
Ethnicity							<0.0001
Hispanic or Latino	08.5 (467)	08.5 (1004)	0	9.8 (5058)	9.4 (19678)	1	
Not Hispanic or Latino/Unknown	91.5 (5023)	91.5 (10784)	0	90.2 (46438)	90.6 (189087)	1	
Race							<0.0001
Black/African American	21.1 (1159)	20.6 (2431)	1	32.9 (16920)	23.0 (48058)	**22**	
White	66.8 (3670)	68.8 (8114)	4	55.1 (28352)	66.3 (138347)	**23**	
Other	12.0 (661)	10.5 (1243)	5	12.1 (6224)	10.7 (22360)	4	
Pain diagnosis							
Abdominal and bowel pain	51.1 (2803)	41.8 (4927)	**19**	47.8 (24590)	40.4 (84345)	**15**	<0.0001
Back pain	69.9 (3840)	67.9 (8007)	4	67.3 (34663)	68.7 (143506)	3	<0.0001
Headache	41.4 (2273)	32.1 (3779)	**19**	41.7 (21457)	30.8 (64277)	**23**	<0.0001
Musculoskeletal chest pain	38.3 (2104)	39.3 (4629)	2	34.8 (17920)	39.0 (81355)	9	<0.0001
Neck pain	41.9 (2300)	35.7 (4209)	**13**	39.5 (20357)	34.8 (72695)	10	<0.0001
Chronic migraine	04.9 (271)	02.1 (251)	**15**	5.3 (2726)	1.8 (3684)	**19**	<0.0001
Tension headache	05.7 (315)	03.7 (441)	9	5.9 (3029)	3.5 (7296)	**11**	<0.0001
Orofacial ear and temporomandibular pain	14.6 (801)	09.4 (1112)	**16**	13.3 (6823)	8.6 (17948)	**15**	<0.0001
Alcohol use disorder	32.8 (1799)	46.2 (5451)	**28**	25.8 (13275)	42.0 (87664)	**35**	<0.0001
Drug use disorder	31.0 (1703)	41.6 (4899)	**22**	23.0 (11831)	33.3 (69493)	**23**	<0.0001
Bipolar disorder	27.0 (1480)	20.7 (2439)	**15**	21.7 (11164)	15.1 (31560)	**17**	<0.0001
General anxiety disorder	24.2 (1331)	19.8 (2334)	**11**	21.4 (11017)	16.2 (33713)	**13**	<0.0001
Major depressive disorder	71.9 (3948)	65.0 (7667)	**15**	68.2 (35123)	57.9 (120846)	**22**	<0.0001
Traumatic brain injury	09.0 (495)	11.9 (1397)	9	7.3 (3756)	11.4 (23773)	**14**	<0.0001
Violent crime/maltreatment	18.9 (1037)	07.0 (825)	**36**	15.9 (8203)	04.2 (8818)	**40**	<0.0001
Military sexual trauma	69.4 (3811)	26.3 (3102)	**96**	70.0 (36051)	16.6 (34541)	**128**	<0.0001
Outpatient visits, mean (SD)	62.4(43.2)	62.5(47.4)	0	51.7 (36.4)	49.2 (41.1)	6	<0.0001

* *p-value across all four groups. Bold text indicates significant ASD values.*

## Discussion

In a large diverse nationwide cohort of Veterans in care, more LGBTQ+ Veterans were diagnosed with PTSD (36.6%) than non-LGBTQ+ Veterans (21.1%). The findings from this study suggest there are demographics, comorbidities, and outpatient care differences between LGBTQ+ Veterans with PTSD and non-LGBTQ+ Veterans with PTSD. The results also suggest an intersectionality of LGBTQ+ status and sex of record in the context of PTSD.

The study’s findings both align with and add to the literature. Overall, these results suggest that issues known to be generally associated with PTSD (i.e., pain, mental health issues, and substance abuse) may be accentuated in some Veteran populations, and while there may be differences among groups for some forms of pain in the wider population, this does not fully explain our study’s findings. For example, rates in transition from episodic to chronic migraine in men and women vary by study (42), with one study (43) finding that the percentage in men was slightly higher (5.4% vs. 4.4%). Individuals with female sex of record in our study reported significantly more chronic migraine. Abdominal/belly pain is associated with PTSD symptom severity and depression in Veterans (13) which may to a degree correlate with the results from our study. As in previous studies (17–19), our study’s findings also suggest that PTSD for some groups may be grounded in aspects outside of combat. Webermann et al. (44) found that one in three women Veterans reports MST and that MST was associated with PTSD. Our findings illustrated a striking difference at the intersection of LGBTQ+ status and sex of record; females compared to males were 3-fold more likely to have MST. Studies on marital status and PTSD have yielded mixed results (45); however, the support of a partner has been shown to mitigate PTSD (46), and in this study, there were significant differences in marital status. TBI was not statistically significant, except in comparing non-transgender male and female sex of record, and non-LGBTQ+ male and female sex of record.

These findings further characterize the PTSD experience for all Veterans and may help guide future research and policy. The findings also suggest tailoring treatment by sex of record, gender identity, sexual orientation minority status, and other factors may be of value and possibly enhance outcomes. Overall, more research is warranted.

### Limitations

Our study has some limitations. Patients were largely male who were in care and may not be generalizable. Because this study relied on EHR data, it was reliant on correct data entry in the recording process. The natural language process could have misclassified Veterans, but this misclassification would have been small and non-differential (37). Additionally, we are unable to identify the sexual orientations and gender identities of transgender Veterans. Transgender status was paired with sex of record, which does not accurately represent the gender identities of these Veterans for multiple reasons. Within the VA, Veterans are permitted to update their sex of record to better align with their gender identity, though this practice is not adopted by all transgender Veterans. We acknowledge that having complete information on the sexual orientations and gender identities of transgender Veterans could provide more detailed data on specific differences within this group. Identifying transgender Veterans in EHR is problematic regardless of the EHR data source (47). Moreover, we did not separate the subgroups of L, G, and B which may have additional informative differences, but that level of granularity is beyond the scope of this study.

### Future work

Future studies will address interventions, and policies regarding PTSD, LGBTQ+ status, and sex of record.

## Conclusion

Veterans, as well as LGBTQ+ individuals and women, are disproportionately at risk for PTSD. In this descriptive statistical analysis, we compared Veterans who had been diagnosed as having PTSD, by LGBTQ+ status and sex of record, with separate analyses specific to transgender status. We found significant differences at the intersection of LGBTQ+ status and sex of record. These findings can potentially enhance future interventions, policy, and research.

## Data Availability

The data analyzed in this study are subject to the following licenses/restrictions: the data include protected health information (PHI), therefore, they are not publicly available.
